# Complete clinical response of metastatic hepatocellular carcinoma to sorafenib in a patient with hemochromatosis: A case report

**DOI:** 10.1186/1756-8722-1-18

**Published:** 2008-10-17

**Authors:** Brian J So, Tanios Bekaii-Saab, Mark A Bloomston, Tushar Patel

**Affiliations:** 1 The Ohio State University Medical Center, 395 W. 12th Avenue, Columbus, OH 43210, USA

## Abstract

Hepatocellular carcinoma is rare, but increasing in prevalence in the United States. Recent studies have shown that sorafenib, a multikinase inhibitor, can reduce tumor progression in patients with this cancer. However, complete remission has not been observed. We report a case of a 78-year old patient with unresectable metastatic hepatocellular carcinoma who had a rapid and complete clinical response following therapy with sorafenib for six months. No evidence of disease recurrence has been noted for 6 months after discontinuation of therapy.

## Background

Hepatocellular carcinoma (HCC) is the third leading cause of cancer death worldwide, with more than 600,000 cases diagnosed every year [1]. Most patients present with advanced and multifocal disease at the time of diagnosis, and the median survival in this setting is less than 6 months. Until recently, there were no effective treatments for advanced HCC [2]. However, a recent phase III randomized, placebo-controlled trial showed an improvement in overall survival in patients with advanced HCC treated with sorafenib compared to placebo controls [3]. Sorafenib has effects on tumor proliferation and angiogenesis. The mechanism of action of sorafenib includes inhibition of multiple kinase targets within the liver. These include members of the Raf family of serine/threonine kinases, and other tyrosine kinases such as Flt-3, kit, Ret, vascular endothelial growth factor receptor 2 (VEGFR-2), vascular endothelial growth factor receptor 3 (VEGFR-3) and platelet-derived growth factor receptor beta (PDGFR-β) [4,5]. The "addiction" of tumors to specific kinases has been demonstrated in some cancers, and targeting these can result in dramatic responses. However, the predominant effect noted with sorafenib has been reduction in tumor progression [3]. We report herein a case of a rapid and complete remission in a patient with the use of sorafenib in metastatic HCC.

## Case Presentation

A 78-year-old man with a 25-year history of hereditary hemochromatosis, with cirrhosis, hypertension, diabetes mellitus, coronary artery disease, and chronic kidney disease (stage II) presented with a six month history of non-productive cough and shortness of breath. The patient also reported an approximately 30-pound weight loss over the previous 4 months. A CT-scan demonstrated a dominant right lobe liver mass and several other intrahepatic lesions as well as multiple lung nodules. PET/CT confirmed that these lesions in the right and left lobes of the liver, as well as a right hilar mass were hypermetabolic. His alpha-feto protein was found to be 13,599 ng/mL. A diagnosis of metastatic hepatocellular cancer was made on the basis of the clinical presentation, imaging studies and elevated tumor markers. His Eastern Cooperative Oncology Group (ECOG) performance status was 2.

Sorafenib therapy was initiated at 400 mg, orally, twice daily dosing. The patient tolerated the therapy well with minimal toxicities, except for the onset of grade 1 diarrhea, which was well controlled medically without dose reduction. A CT scan of the liver obtained after one month of therapy showed a significant reduction of tumor-burden with a decrease in the size of the mass in the right lobe of the liver from 4.5 × 5.0 cm to 3.9 × 4.3 cm. Similarly, a CT scan of the chest revealed almost complete resolution of the pulmonary nodules, with only one 0.4 cm nodule in the right apex. Concomitantly, there was a dramatic reduction in the patient's alpha-fetoprotein to 4.5 ng/mL. The patient's follow-up visit the following month continued to show that the patient was tolerating his medication well. His functional status continued to improve (ECOG 1). A repeat CT-scan of the abdomen after 5 months showed further reduction in the liver tumor, with a lesion in the right lobe measuring only 2.6 × 3.4 cm, and a repeat PET-scan did not show any evidence of a hypermetabolic lesion in the liver 1. The patient continued sorafenib for a total 6-month course, and subsequent alpha-fetoprotein monitoring was performed to assess for sustained response 2. At follow-up six months after completion of the sorafenib treatment, there was no clinical evidence of recurrence, alpha-fetoprotein remained within normal limits and CT imaging continued to show evidence of complete remission.

**Figure 1:**
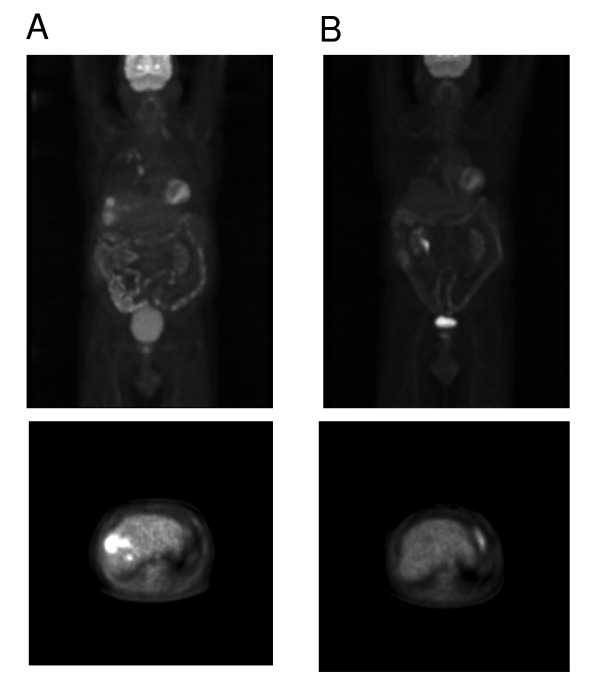
**Positron Emission Tomography imaging scan prior to and following therapy.** The top panels represent a coronal view whereas the bottom panels represent a horizontal section through the liver. (A) Pre-treatment imaging shows diffuse uptake in the liver, and lungs. (B) Subsequent imaging after 20 weeks of therapy with sorafenib reveals loss of uptake in these regions consistent with a dramatic reduction on tumor burden.

**Figure 2:**
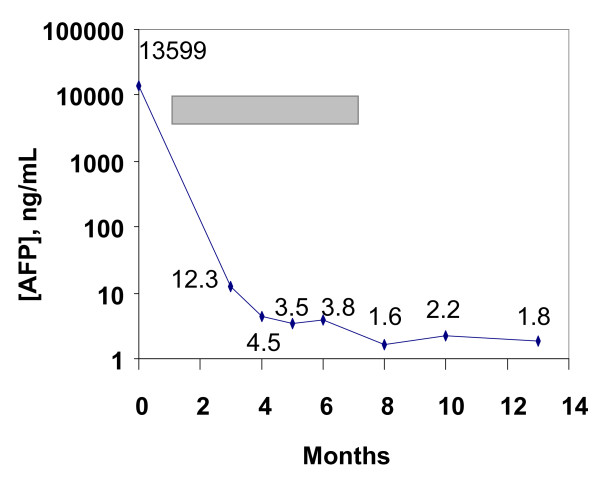
**Temporal changes in alpha-feto-protein expression.** The duration of treatment with sorafenib is indicated in the gray bar. Initiation of sorafenib resulted in a dramatic reduction in serum AFP levels.

## Conclusion

Advanced metastatic HCC continues to portend a poor prognosis with few therapeutic options [2]. Sustained complete remission has been reported in metastatic HCC only following aggressive surgical resection and cytotoxic chemotherapy. This case represents the first known complete response to sorafenib in metastatic HCC.

The SHARP (Sorafenib HCC Assessment Randomized Protocol) trial was instrumental in showing the efficacy of sorafenib on the overall survival of patients with HCC. An increase of 44% in overall survival was seen, and a time-to-progression of disease of 5.5 months, when compared to 2.8 months seen in the placebo group. Of the 299 patients randomized to receive sorafenib therapy in this study, none showed complete response, 7 (2.3%) showed partial response, and the majority, 211 (71%) showed stable disease [3]. Similarly complete remission has not been observed in clinical trials of sorafenib in patients with renal cell carcinoma. Preliminary studies have shown an anti-tumor activity of sorafenib in a variety of tumor types such as renal cell carcinoma, melanoma, thyroid cancer, ovarian cancer, sarcoma, and pancreatic cancer. None of these tumor types is characterized by elevated alpha-fetoprotein or imaging characteristics observed in our patient.

The case illustrates a patient who had a rapid and complete response to sorafenib therapy, based on imaging and tumor marker response. The response to therapy was unlike that seen in reported clinical trials of sorafenib. In the SHARP trial, 83% of the 902 patients in the study had protocol deviation due to a variety of reasons. The original dosing of sorafenib called for 400 mg, twice daily dosing. Our patient was able to tolerate the complete dose throughout the entire 6 month course without interruption or dose reduction. While the mechanism of response in our patient is unclear, it is conceivable that his tumor was strongly dependent for survival on one or more of the receptor tyrosine kinases that are inhibited by sorafenib. Although this phenomenon has been noted in some other solid tumors such as gastrointestinal stromal tumors, we do not have any direct evidence of this in our patient. Moreover, it is unknown how the patient's underlying hemochromatosis may have contributed to this response due to the lack of reported experience with treatment of HCC in this condition.

In conclusion, this case demonstrates that dramatic therapeutic responses are possible with the use of sorafenib for the treatment of metastatic HCC. Our patient's apparent complete response is certainly unusual. We speculate that a subset of patients capable of attaining a complete remission will be identified as more patients with advanced metastatic HCC undergo therapy with sorafenib. The durability of such a robust response is yet to be determined.

## Competing interests

The authors declare that they have no competing interests.

## Authors' contributions

The original manuscript was written by BS. All authors participated in drafting and editing the manuscript. All authors read and approved the final manuscript.

## Authors' information

The authors provide specialized, multidisciplinary clinical care for patients with Hepatocellular Cancer at the Ohio State University Comprehensive Cancer Center – James Cancer Hospital.

## Consent

The patient has provided informed consent for the publication of this case report and accompanying images.
